# Randomized controlled trials in children’s heart surgery in the 21st century: a systematic review

**DOI:** 10.1093/ejcts/ezx388

**Published:** 2017-11-23

**Authors:** Nigel E Drury, Akshay J Patel, Nicola K Oswald, Cher-Rin Chong, John Stickley, David J Barron, Timothy J Jones

**Affiliations:** 1 Department of Paediatric Cardiac Surgery, Birmingham Children’s Hospital, Birmingham, UK; 2 Institute of Cardiovascular Sciences, University of Birmingham, Birmingham, UK; 3 Department of Physiology, Anatomy and Genetics, University of Oxford, Oxford, UK

**Keywords:** Systematic review, Clinical trials, Paediatric cardiac surgery, Evidence-based medicine

## Abstract

**OBJECTIVES:**

Randomized controlled trials are the gold standard for evaluating health care interventions, yet are uncommon in children’s heart surgery. We conducted a systematic review of clinical trials in paediatric cardiac surgery to evaluate the scope and quality of the current international literature.

**METHODS:**

We searched MEDLINE, CENTRAL and LILACS, and manually screened retrieved references and systematic reviews to identify all randomized controlled trials reporting the effect of any intervention on the conduct or outcomes of heart surgery in children published in any language since January 2000; secondary publications and those reporting inseparable adult data were excluded. Two reviewers independently screened studies for eligibility and extracted data; the Cochrane Risk of Bias tool was used to assess for potential biases.

**RESULTS:**

We identified 333 trials from 34 countries randomizing 23 902 children. Most were early phase (313, 94.0%), recruiting few patients (median 45, interquartile range 28–82), and only 11 (3.3%) directly evaluated a surgical intervention. One hundred and nine (32.7%) trials calculated a sample size, 52 (15.6%) reported a CONSORT diagram, 51 (15.3%) were publicly registered and 25 (7.5%) had a Data Monitoring Committee. The overall risk of bias was low in 22 (6.6%), high in 69 (20.7%) and unclear in 242 (72.7%).

**CONCLUSIONS:**

The recent literature in children’s heart surgery contains few late-phase clinical trials. Most trials did not conform to the accepted standards of reporting, and the overall risk of bias was low in few studies. There is a need for high-quality, multicentre clinical trials to provide a robust evidence base for contemporary paediatric cardiac surgical practice.

## INTRODUCTION

Congenital heart disease is the commonest type of congenital anomaly, affecting 12 children born in the UK every day [1]. There is a marked heterogeneity of structural cardiovascular defects, often leading to cyanosis or heart failure and requiring complex surgery in early childhood. Despite these challenges, advances in paediatric cardiac surgery have been rapid, founded on the work of visionary pioneers and improved incrementally through adjustments in operative technique, supported by developments in imaging, anaesthesia, intensive care, surgical technology and hybrid approaches to management. Surgery is a high-tech, high-stakes intervention that radically alters the natural history of congenital heart disease, especially for those at an early stage in their life trajectory with the most to gain. Today, surgeons can offer the prospect of ‘cure’ or successful palliation in almost all structural congenital heart conditions, yet the impact of surgical techniques and decision-making on long-term survival, reintervention, cardiac function, exercise tolerance and quality of life is not fully understood [2].

Randomized controlled trials (RCTs) represent the gold standard in evaluating health care interventions through rigorous testing of a predefined protocol with minimization of bias [3]. However, much of modern paediatric cardiac surgical practice is based on expert opinion, institutional case series or experimental evidence derived from adults [4]. We therefore conducted a systematic review of all RCTs assessing the outcomes of heart surgery in children to identify the scope and quality of the current literature.

## MATERIALS AND METHODS

This review was conducted with reference to the Cochrane Handbook for Systematic Reviews [5, 6] and reported in accordance with the PRISMA statement [7], where appropriate. All eligibility criteria, search terms and data items were prespecified.

### Trial eligibility

All RCTs and quasi-RCTs reporting the effect of any intervention on children undergoing cardiac surgery or their families published in any language since 1 January 2000 were included. The definition of a child was based on the authors’ characterization, and cardiac surgery was defined as a therapeutic clinical procedure on the heart or central vasculature, performed wholly or in part by a cardiac surgeon, with or without the use of cardiopulmonary bypass.

Trials were excluded if the outcome measures were not directly related to the conduct or outcomes of surgery, such as trials specifically and solely related to anaesthesia, analgesia, nutrition, physiotherapy, pharmacokinetics, transplant immunosuppression or weaning from ventilation. Invasive life support including extracorporeal membrane oxygenation was excluded unless performed as part of a primary cardiac surgical procedure. Trials including both adults and children were only included if the publication presented the paediatric data separately. Secondary publications, sub-studies and long-term outcomes of previously reported trials were excluded, unless the results were specifically related to cardiac surgery while the original was not. Trials published only as a conference abstract or for which all options to obtain the full text were exhausted were excluded due to insufficient data.

### Search strategy

We searched international primary research databases (PubMed, CENTRAL and LILACS) from 1 January 2000 to 31 August 2016 and reference lists of relevant articles and systematic reviews to identify all eligible studies. We combined validated search strategies to identify RCTs, studies including children and those involving cardiac surgery (see Supplementary Material, Search Strategies). For example, to identify the RCTs in PubMed, we used the Cochrane Highly Sensitive Search Strategy for identifying randomized trials: sensitivity- and precision-maximizing version [5] and to identify studies including children, we adapted the improved Cochrane Childhood Cancer Group filter for PubMed developed by Leclercq *et al.* [8].

### Study selection and data extraction

Abstracts and full-text publications of identified articles were screened independently by two reviewers (N.E.D. and A.J.P.) to generate a database of included studies. Data were extracted independently by two reviewers (two of N.E.D., A.J.P. and N.K.O.) from the full-text publication and any published protocols or supplemental material; assessments in previous systematic reviews were corroborated. A full list of data items and definitions is available in the Supplementary Material, Data items and definitions. Any discrepancies were resolved by consensus. Non-English language articles were evaluated in conjunction with individuals with a clinical or research methodology background and fluent in that language (C-RC for Chinese, see Acknowledgements for others).

### Risk-of-bias assessment

The Cochrane Risk of Bias Tool was used to assess the methodological quality of eligible trials in 8 domains: sequence generation; allocation concealment; blinding of participants, personnel and outcome assessors; incomplete outcome data; selective outcome reporting; and other potential threats to validity [6]. Trials were rated as low, unclear or high risk of bias for each factor; the overall risk of bias was determined for each trial as low (low risk in all domains), high (high risk in one or more domains) or unclear (neither of the above).

### Statistical analysis

Statistical analysis was performed using *R* (https://www.r-project.org/). All continuous data were expressed as medians with interquartile ranges (IQRs) and categorical data as counts and percentages where relevant. The Fisher’s exact test was used to compare categorical variables, and linear regression was used to model the relationship between the number of children randomized per trial and the year of publication. Significance testing was 2 sided with the significance level determined at *P* < 0.05.

## RESULTS

**Figure 1:**
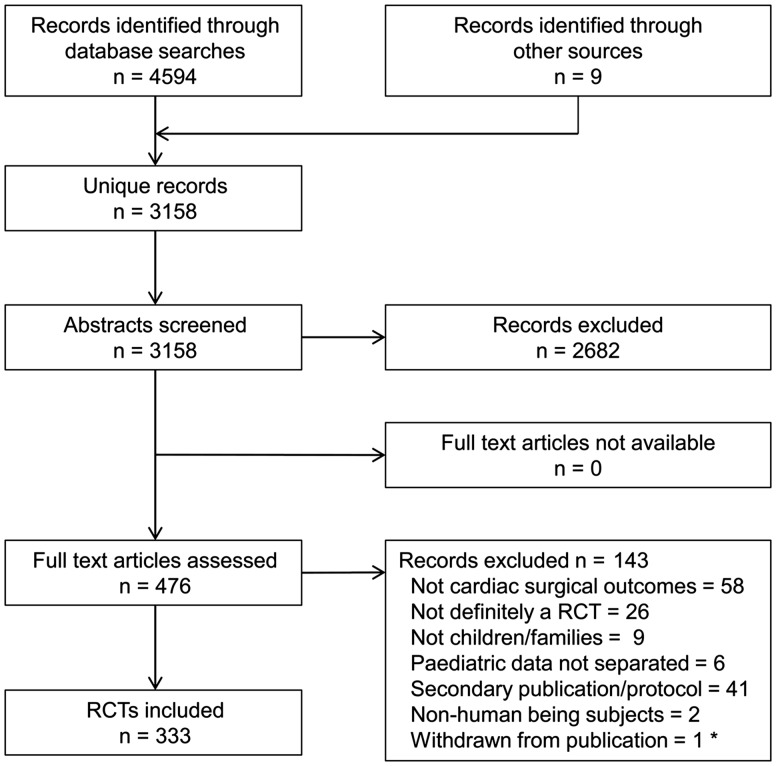
Study selection. *Retracted by the Editor. RCT: randomized controlled trial.

From 3158 unique records, we identified 333 RCTs published since 1 January 2000, randomizing a total of 23 902 children undergoing surgery. The flow of studies through the systematic review process is documented in Fig. 1. All full-text articles were sourced online, via national libraries or directly from the authors.

**Figure 2:**
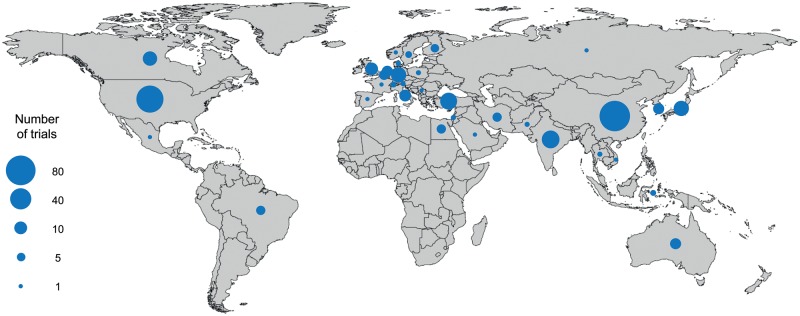
World map of published trials by country of origin. Multinational trials are counted in each contributing country.

Characteristics of the included trials are listed in Table 1. The majority of the studies were single-centre studies (318, 95.5%), with only 7 (2.1%) trials conducted across more than 3 sites. Trials originated from 34 countries (Fig. 2), with China (84, 25.2%), USA (65, 19.5%), India (21, 6.3%), Turkey (20, 6.0%) and Japan (17, 5.1%) being the most frequent; only 5 (1.5%) trials were multinational, conducted in 2 or 3 countries. Forty-three (12.9%) trials were published in a language other than English: 35 (10.5%) in Chinese, 2 (0.6%) each in Korean and Turkish and 1 (0.3%) each in German, Persian, Portuguese and Russian. Trials were most commonly published in specialist cardiothoracic surgery journals (120, 36.0%), with few reaching high-impact cardiovascular [9, 2.7%: *Circulation* (5) and *Journal of the American College of Cardiology* (4)] or general medical journals [6, 1.8%: *Lancet* (3) and *New England Journal of Medicine* (3)]. The majority of studies were Phase II (313, 94.0%), with only 15 (4.5%) Phase III or IV trials; however, these late-phase trials were significantly more likely to be published in high-impact journals (5/15 vs 10/318, *P* = 0.0001).

**Table 1:** Characteristics of the included trials

Characteristics	*n* (%)	Characteristics	*n* (%)
Late phase (III or IV)	15 (4.5)	Source of funding	
Multinational	5 (1.5)	Funding reported	138 (41.1)
Multicentre	15 (4.5)	Public^a^	75 (22.5)
Continent of origin		Institutional^a^	43 (12.9)
Asia	147 (44.1)	Charity/private^a^	30 (9.0)
Europe^a^	92 (27.6)	Commercial^a^	16 (4.8)
North America^a^	75 (22.5)	None	16 (4.8)
Oceania	8 (2.4)	Not reported	179 (53.8)
Africa	6 (1.8)	Outcomes	
South America	6 (1.8)	Clinical	123 (36.9)
Language of publication		Surrogate	144 (43.2)
English	290 (87.1)	Combined^a^	66 (19.8)
Chinese	35 (10.5)	Economic^a^	1 (0.3)
Other	8 (2.4)	Randomization
Design		Simple unrestricted	87 (26.1)
Parallel groups	314 (94.3)	Block/stratified	61 (18.3)
Factorial	16 (4.8)	Minimization	5 (1.5)
Crossover	3 (0.9)	Other technique	2 (0.6)
Number of arms		Quasi- or non-random^b^	17 (5.1)
2	279 (83.8)	Unclear	161 (48.3)
3	41 (12.3)	Explicit call for larger trial to answer the same question	89 (26.7)
4 or more	13 (3.9)		

a Includes multiple counting.

b Such as allocation by alternation, hospital number or site.

**Figure 3:**
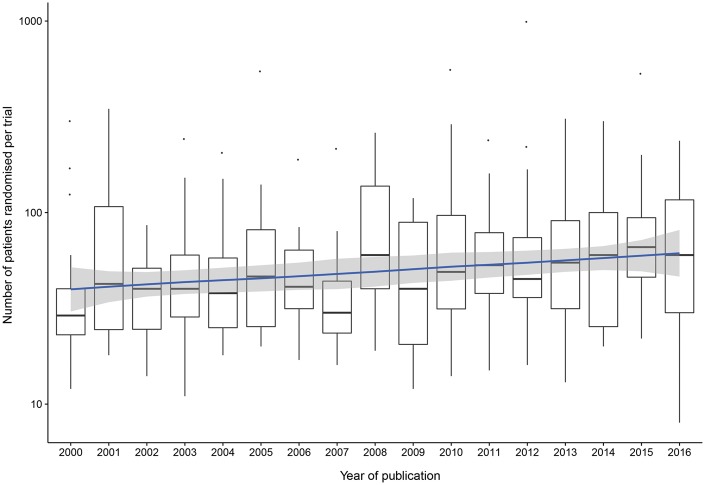
Logarithmic Tukey box plot of the number of children randomized per trial by year. Linear regression line (*R*^2^ 0.026, *P* = 0.003) suggests no meaningful change over time.

In all trials, the subject of the intervention was the child undergoing surgery. The number of children randomized ranged between 8 and 989 with a median of 45 patients (IQR 28–82); 17 (5.1%) trials analysed 100 or more patients per arm, with an average of 1 trial per year worldwide. The number of children randomized per trial by year is shown in Fig. 3; linear regression showed a slight increase in the median number of patients per trial over time (*R*^2^ 0.026, *P* = 0.003). Recruitment rate was reported in 59 (17.7%) articles, with a median of 90% (IQR 72–96) of eligible children recruited, and trial duration was reported in 184 (55.3%), recruiting over a median of 18 (IQR 12–29) months. Of those trials reporting a power calculation, 95 (87.2%) achieved the target number of patients and 26 (7.8%) had a dropout rate of 10% or more between randomization and analysis of the primary outcome.

The leading topics of investigation were cardiopulmonary bypass techniques/components (92, 27.6%), myocardial protection strategies (60, 18.0%) and blood transfusion/conservation methods (45, 13.5%). Drugs (171, 51.4%), cardiopulmonary bypass techniques (78, 23.4%) and other techniques (43, 12.9%) were the most frequently studied interventions (Table 2); comparison of 2 surgical approaches (6, 1.8%) or of a surgical technique with an interventional catheter procedure (5, 1.5%) was less commonly evaluated. A primary outcome measure was defined in 193 (58.0%) trials and of these, 103 (53.4%) trials were clinical; surrogate end-points only were reported in 144 (43.2%) trials. Mortality was defined as an end-point in 12 (3.6%) studies, but only 1 (0.3%) study demonstrated a survival difference between groups [9].

**Table 2:** Interventions studied

Interventions	*n* (%)	Interventions	*n* (%)
CPB technique	78 (23.4)	Drug	171 (51.4)
Ultrafiltration^a^	22 (6.6)	Protease inhibitor	25 (7.5)
Perfusion^a^	20 (6.0)	Corticosteroids^a^	23 (6.9)
Prime	18 (5.4)	Cardioplegia^a^	19 (5.7)
Temperature^a^	8 (2.4)	Pulmonary vasodilator	17 (5.1)
Reoxygenation	6 (1.8)	Other vasoactive	15 (4.5)
Haemodilution	4 (1.2)	Natural remedy^a^	15 (4.5)
Other technique	43 (12.9)	Anaesthetic agent	13 (3.9)
Ischaemic conditioning	14 (4.2)	Antiarrhythmic Thyroid hormone	6 (1.8) 6 (1.8)
Surgical	6 (1.8)	Recombinant factor	5 (1.5)
Surgery vs interventional	5 (1.5)	ACE inhibitor Insulin	4 (1.2) 3 (0.9)
Drainage	4 (1.2)	Other drug	20 (6.0)
Rewarming	4 (1.2)	Blood/fluid administration	20 (6.0)
Surgical access	3 (0.9)		
Other technique	7 (2.1)	Blood product^a^	17 (5.1)
Device	22 (6.6)	Intravenous fluid	3 (0.9)
CPB circuit^a^	13 (3.9)	Miscellaneous	4 (1.2)
Haemostasis management	4 (1.2)	Music therapy Other	2 (0.6) 2 (0.6)
Cell saver	2 (0.6)		
Other device	3 (0.9)		

a Comparisons between interventions are counted in each category.

ACE: angiotensin-converting enzyme; CPB: cardiopulmonary bypass.

### Quality of trials

**Figure 4:**
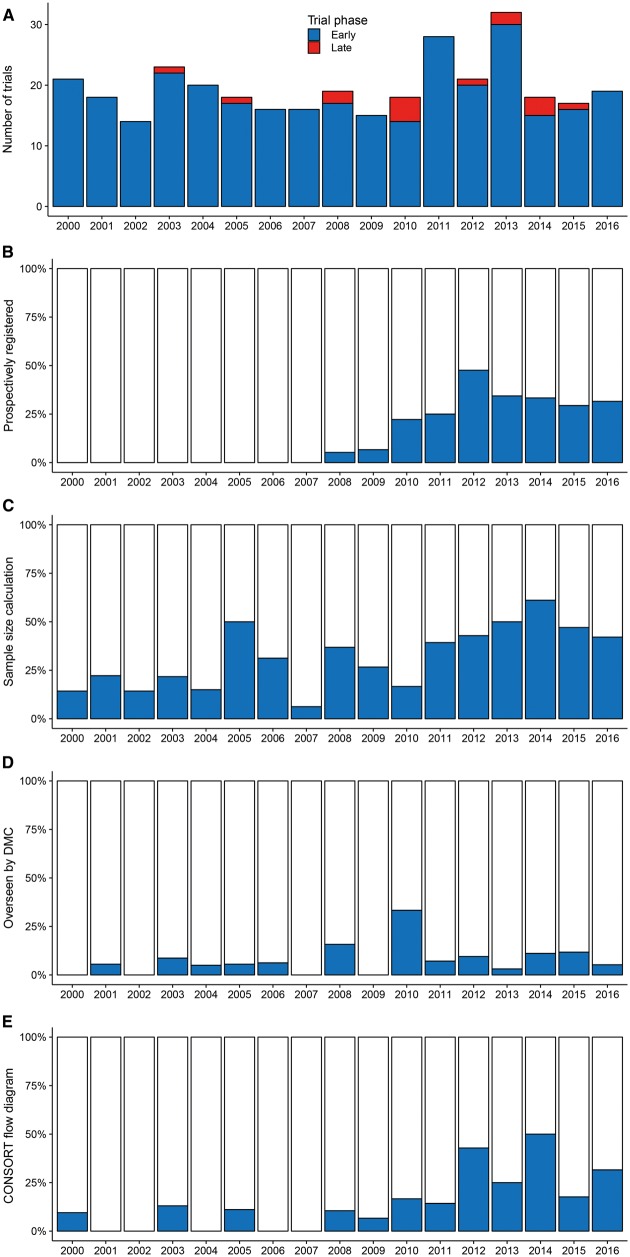
Histograms of trial characteristics by year. (**A**) Published, by phase; (**B**) prospectively registered on a publicly accessible trial database; (**C**) reporting a sample size calculation; (**D**) overseen by a Data Monitoring Committee (DMC) and (**E**) publishing a CONSORT flow diagram.

Regarding standards for the conduct and reporting of clinical trials [3], 51 (15.3%) were prospectively registered on a publicly accessible trial database, 109 (32.7%) performed a sample size calculation, 25 (7.5%) were scrutinized by an independent Data Monitoring Committee (DMC) and 52 (15.6%) published a CONSORT flow diagram (Fig. 4). An explicit intention-to-treat analysis was performed in 45 (13.5%) trials, a trial protocol published in 9 (2.7%) and a Clinical Trials Unit or trial network involved in 8 (2.4%). Nine (2.7%) trials were stopped early: 3 on grounds of futility, 2 due to harm or side effects, 1 due to benefit and 3 for logistical reasons (drug withdrawn or staff changes). One (0.3%) trial was suspended by the DMC on safety grounds but restarted with an amended protocol and 2 (0.6%) were extended, either by the DMC or by the regulator. Trials with independent oversight were more likely to be stopped early or suspended (4/25 vs 6/308, *P* = 0.004).

**Figure 5:**
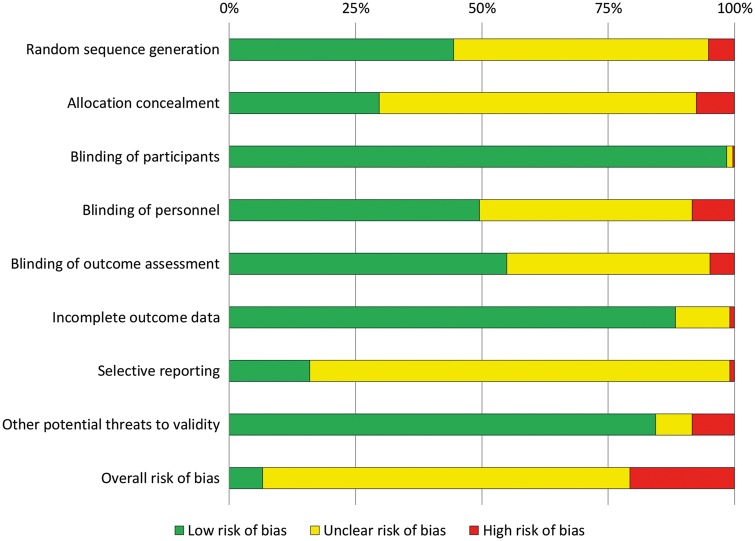
Risk of bias.

Risk-of-bias assessment for each of the 8 domains and overall is shown in Fig. 5. The overall risk of bias was low for 22 (6.6%), high for 69 (20.7%) and unclear for 242 (72.7%) trials; the high proportion of unclear resulted from poor reporting of randomization and masking procedures and an inability to exclude selective reporting due to a lack of trial registration or published protocol. All trials with a low risk of bias across all domains were published since 2008 and were more likely to be Phase III, multicentre and conducted in Europe, North America or Australia (all *P* < 0.005).

## DISCUSSION

Randomized controlled trials only represent the gold standard when appropriately designed, conducted and reported [3]. Although Phase II efficacy and safety trials have a role in determining whether potential treatments are worth pursuing, it is Phase III clinical effectiveness trials that influence guidelines and change practice. In this systematic review of the recent literature, we identified 333 RCTs in children’s heart surgery from 34 countries reported since the turn of the millennium. These were mostly single-centre, Phase II trials that were not prospectively registered, recruited small numbers of patients without independent oversight and were not reported to international standards [3, 10, 11]. Few studies dissected the merit of the surgical interventions, instead focused on adjuncts such as cardiopulmonary bypass or myocardial protection and examined surrogate end-points that were susceptible to systematic bias. There was no meaningful increase in the number of patients randomized per trial over time, yet this did not appear to reflect difficulties in recruitment: a median of 90% of eligible patients were recruited; target sample size was achieved in 87% of studies and none was stopped early due to poor recruitment.

Our findings are typical of the paediatric trials literature. Hamm *et al*. [12] reported that only 12% of paediatric trials declared prospective trial registration, fewer than 5% were overseen by a DMC and the risk of bias was low in 8% but high in 59% of trials. Similarly, Duffett *et al.* [13] found that half of paediatric critical care trials randomized fewer than 50 patients, most focused on intermediate or surrogate outcomes, 43% reported a target sample size, 15% were prospectively registered and the overall risk of bias was low in only 4%. Furthermore, Shamliyan and Kane [14] reported that of interventional studies involving children registered on clinicaltrials.gov, only 29% of completed studies were published in peer-reviewed journals, revealing a substantial publication bias and implying that these may be just the tip of a low-value, low-quality trial iceberg.

Contrary to adults with acquired disease, children with congenital heart disease present a range of complex anatomical and physiological challenges that may require surgery at any stage of development from preterm neonate to post-pubertal adolescent. The immature heart, brain and other organs are often exposed to chronic hypoxia prior to surgery, which may alter the ability to cope with intraoperative or perioperative perturbations in blood flow and oxygen delivery. Subsequent growth, or lack of it, may significantly impact on the need for surgical or catheter reinterventions, such as balloon dilatation, upsizing of conduits or valve replacement. It is a founding principle of paediatrics that ‘children are not just small adults’, yet many aspects of current practice have been extrapolated from adult studies, using ‘hand-me-down’ paradigms with little or no paediatric data to support their application [15–18]. Surgical trials in children are considered more challenging due to a number of factors including: few cases per centre, resulting from the low disease burden and heterogeneity of conditions; complex procedures with marked variations in practice between surgeons and institutions; low mortality rates yet a lack of other validated clinical outcome measures; greater scrutiny from ethics committees and regulators; the higher cost of paediatric trials; and a perceived lack of willingness of surgeons and parents to enter their children into randomized trials [4, 19]. These barriers predispose to inadequate power leading to inconclusive results, increased uncertainty and ineffective use of resources.

To address these concerns, the Paediatric Heart Network (PHN) was established in 2001 by the National Heart, Lung and Blood Institute, using an investigator-led model of core clinical centres and auxiliary sites across the USA and Canada [4, 17]. Policies and procedures are developed by a Central Steering Committee, with representation from all participating centres, and implemented by a Data Coordinating Centre, with scientific and safety oversight provided by Protocol Review Committee and DMC, respectively. This explicit engagement of clinical investigators coupled with central investment in personnel and infrastructure have been fundamental to their success. The landmark Single Ventricle Reconstruction (SVR) trial randomized 555 infants with single-ventricle lesions (84% of those eligible) to the Norwood procedure, with either a modified Blalock–Taussig shunt or a right ventricle to pulmonary artery shunt across 15 sites in just over 3 years [9]. This trial demonstrated not only the power of collaboration to increase sample size in a rare condition but also that surgeons and parents were willing to randomize children to different treatments, even in high-risk complex neonatal cardiac surgery, when there is clinical equipoise [17]. To date, it has produced more than 20 publications including a publicly available data set and remains the only Phase III trial in children’s heart surgery to demonstrate a difference in 1-year transplant-free survival [9]; perhaps, more importantly, this difference was lost by 3 years [20], and further follow-up is keenly awaited. Unfortunately, despite being uniquely positioned to conduct such trials, the SVR trial remains the only surgical interventional trial of the PHN to be completed, and no similar studies are currently recruiting [21].

Of the trials identified in this review, 10 (3.0%) were conducted in the UK. All were single-centre Phase II trials, randomizing 431 patients in total, or less than 1% of the approximately 65 000 children who underwent cardiac surgery in the National Health Service (NHS) over the same period [22]. In contrast, more than 70% of children diagnosed with cancer in the UK are currently enrolled into national or international Phase III clinical trials [23]; this approach has played a key role in the dramatic increase in survival of childhood cancers over the past 50 years, and providing the opportunity to participate in such a trial is now considered best practice [24]. With national commissioning of specialist services and cradle-to-grave follow-up, plus access to National Institute of Health Research infrastructure and well-developed Clinical Trials Units, the modern NHS should be an ideal environment to conduct multicentre RCTs in children’s heart surgery. However, in the wake of the Bristol Royal Infirmary Inquiry of 2001 [25], the focus of UK paediatric cardiac surgical centres was on maintaining and improving standards of direct patient care; research has been a secondary consideration and has since lagged behind other specialities. The new Congenital Heart Disease review of services in England intends to centralize clinical care into fewer, larger Specialist Children’s Surgical Centres within regional networks and deems participation in research to be an essential activity [26]. Indeed, 2 recent multicentre observational studies, the Infant Heart Study and the Cardiac Morbidity Study [27, 28], have demonstrated an appetite for collaboration in the UK, and both congenital heart disease and cardiovascular surgery have been identified by the British Heart Foundation as key elements of their research funding strategy [1].

The outcomes of cardiac surgery in children have improved markedly over the recent decades [2], yet continued improvement will require a team science approach with robust, multicentre, Phase III clinical trials central to providing the evidence base for clinical practice. There remain many important unanswered questions, such as surgical valvotomy versus balloon valvuloplasty for aortic stenosis or comparing types of right ventricle to pulmonary artery conduit, and these are better addressed by late-phase trials rather than endless cycles of pro–con expert debates. A solution to the lack of such trials both in the UK and elsewhere is the development of specific national congenital heart disease research networks, following a similar model to the PHN, with the potential for large-scale international collaborations. The SVR trial has set the standard for the evaluation of surgical interventions in congenital heart disease [4, 9]; this review suggests that in the design, conduct and reporting of future trials, we should:

- Identify key research questions of genuine clinical equipoise that are of the greatest importance to all stakeholders including children, parents, clinicians, researchers, charities, funders and industry.
- Obtain preliminary data from observational studies, disease-specific registries and nationally reported data sets to assess feasibility and identify potential outcome measures [4, 28].
- Select a validated, standardized, clinically important primary end-point as part of a minimum core outcome set for congenital heart disease to facilitate meta-analyses of pooled data [29].
- Ensure an adequate sample size and timely recruitment through an inclusive, collaborative approach to multicentred trials with investment from local investigators and patient organizations through all stages of the trial.
- Identify *a priori* subgroups that may show differential responses to interventions, such as neonates or those with chronic hypoxaemia, ensuring sufficient power to avoid Type II errors [16].
- Utilize existing Clinical Trial Units and Clinical Research Network infrastructure to facilitate efficient multisite set-up and delivery.
- Conduct and report the trial rigorously to international standards [3, 11, 30], minimizing potential biases and upholding the quality of data and outputs.
- Provide oversight by an independent DMC to ensure the safety of participants and the integrity of trial data [16].
- Facilitate ancillary studies and public data sharing to maximize the value of trial investment [4].
- Engage and educate the wider community through user involvement and understand their perspectives on research involving children to improve the design and conduct of trials.

### Strengths and limitations

The strengths of this systematic review include the comprehensive search strategy, independent review procedures and obtainment of the full text of all potential articles in all languages. The limitations include a risk of reporting bias, although unpublished studies would be expected to be less well conducted, and limiting the scope to RCTs, which are not the only source of valuable evidence to inform clinical practice.

## CONCLUSION

‘Lack of research, poor research, and poorly reported research are violations of children’s human rights’—Richard Horton, plenary address to 1st StaR Child Health summit 2009 [11].

In conclusion, this systematic review demonstrates that the recent literature in paediatric cardiac surgery contains few late-phase clinical trials. Most published trials are small, single-centre studies of low value and uncertain quality, providing a limited evidence base for contemporary practice. As a congenital heart disease community, we have the responsibility to provide scientific leadership and work together to conduct well-designed, rigorously conducted, multicentre clinical trials with clearly defined, clinically relevant end-points that answer important questions to improve the outcomes of surgery for our patients and their families. Our colleagues in other specialties, such as paediatric oncology [23], have made the opportunity to participate in late-phase clinical trials part of the routine care pathway and so must we—*carpe diem*.

## SUPPLEMENTARY MATERIAL

Supplementary material is available at *EJCTS* online.

## Supplementary Material

Supplementary Data
