# Supplementary material for: Randomized controlled trials in children’s heart surgery in the 21st century: a systematic review
Source: Eur J Cardiothorac Surg. 2017 Nov 23;53(4):724–31. doi: 10.1093/ejcts/ezx388 (PMC5848812; doi:10.1093/ejcts/ezx388)
Supplement: Supplementary Data [file supplementary_material_ezx388.docx]

**SUPPLEMENTARY MATERIAL**

**Search strategies**

PubMed

#1 randomized controlled trial [pt] OR controlled clinical trial [pt] OR randomized [tiab] OR placebo [tiab] OR clinical trials as topic [mesh: noexp] OR randomly [tiab] OR trial [ti]

#2 animals [mh] NOT humans [mh]

#3 #1 NOT #2 †

#4 infan* OR newborn* OR new-born* OR perinat* OR neonat* OR baby* OR babies OR girl* OR boy* OR kid OR kids OR child* OR paediatrics [mh] OR pediatric* OR paediatric* OR peadiatric* OR prematur* OR preterm* OR congenital* ‡

#5 cardiac surgery OR heart surgery OR cardiothoracic surgery OR cardiovascular surgery

#6 #3 AND #4 AND #5

Filters: Publication date from 2000/01/01 to 2016/10/31.

*mh*, Medical Subject Heading (MeSH) term (‘exploded’); *mesh: noexp*, Medical Subject Heading (MeSH) term (not ‘exploded’); *pt*, Publication Type; *ti*, title; *tiab*, title or abstract.

† Cochrane Highly Sensitive Search Strategy for identifying randomized trials in MEDLINE: sensitivity- and precision-maximizing version (2008 revision); PubMed format.^1^

‡ adapted from Improved CCG child filter for PubMed.^2^

CENTRAL

Cochrane Central Register of Controlled Trials: Issue 9 of 12, September 2016

#1 cardiac surgery OR heart surgery OR cardiothoracic surgery OR cardiovascular surgery

#2 child* OR infan* OR neonat* OR pediatric* OR paediatric*

#3 #1 AND #2

Publication Year from 2000 to 2016

LILACS

#1 ((pt:"randomized controlled trial" OR pt:"controlled clinical trial" OR pt:"multicenter study" OR mh:"randomized controlled trials as topic" OR mh:"controlled clinical trials as topic" OR mh:"multicenter studies as topic" OR mh:"random allocation" OR mh:"double-blind method" OR mh:"single-blind method") OR ((ensaio$ OR ensayo$ OR trial$) AND (azar OR acaso OR placebo OR control$ OR aleat$ OR random$ OR enmascarado$ OR simpleciego OR ((simple$ OR single OR duplo$ OR doble$ OR double$) AND (cego OR ciego OR blind OR mask))) AND clinic$)) AND NOT (mh:animals OR mh:rabbits OR mh:rats OR mh:primates OR mh:dogs OR mh:cats OR mh:swine OR pt:"in vitro") §

#2 tw:(cardiac surgery OR heart surgery OR cardiothoracic surgery OR cardiovascular surgery) OR mh:"Cardiac Surgical Procedures" OR mh:“Heart Defects, Congenital”

#3 mh:(“infant” OR “child, preschool” OR “child”) OR preescolar$ OR pré-escolar$ OR niño$ OR criança$ OR infant$ OR lactante$ OR child$ OR pediatric$ OR paediatric$

#4 #1 AND #2 AND #3

Filter: Publication Year from 2000 to 2016

*mh*, Descriptores en Ciencias de la Salud (DeCS)/Medical Subject Heading (MeSH) terms; *pt*, Publication Type; *tw*, Text Words.

§ Highly sensitive search strategy for clinical trials in Literatura Latino Americana e do Caribe em Ciências da Saúde (LILACS).^3^

**Data items and definitions**

Trial descriptors

Name, initials of the first author (text)

Name of the journal in which it was published (text)

Year of publication (numerical)

PubMed Identifier (PMID), if applicable (numerical)

Number of secondary publications identified (numerical)

Language of publication (categorical)

Trial demographics

Number of centres at which patients underwent surgery (numerical)

Name(s) of centres at which patients underwent surgery (text)

Number of countries in which patients were recruited (numerical)

Name(s) of countries in which patients were recruited (text)

Was a Clinical Trials Unit or central coordinating centre involved? (Yes/No)

Was a CONSORT flow diagram included in the publication (Yes/No)

Was a trial protocol published previously (Yes/No)

Was the trial registered on a publicly-accessible database? (Yes/No)

If so, trial registration number (numerical)

Number of participants randomised (numerical)

Number of participants included in analysis of the primary outcome (numerical)

Recruitment rate, number randomised/number eligible (percentage)

Number of months over which recruitment took place (numerical)

Was the trial externally funded? (Yes/No)

Type(s) of organisation who funded the trial (categorical)

Target of the intervention eg. child, parent, family (categorical)

Mean age of participants (numerical)

Congenital heart condition(s) included (categorical)

Trial design

Phase of trial (categorical)

Design of trial eg. parallel, factorial, cross-over (categorical)

Number of arms to which participants could be allocated (numerical)

Type of randomisation eg. simple, block stratified (categorical)

Was a sample size calculation reported? (Yes/No)

Intervention under investigation (text)

Type of intervention under investigation (categorical)

Was oversight provided by an independent body eg. Data Monitoring Committee? (Yes/No)

Cochrane Risk of Bias Tool ^4^

Randomisation sequence (low, unclear, high)

Allocation concealment (low, unclear, high)

Blinding participants (low, unclear, high)

Blinding personnel (low, unclear, high)

Blinding outcome assessors (low, unclear, high)

Incomplete outcomes (low, unclear, high)

Selective reporting (low, unclear, high)

Other bias (low, unclear, high)

Outcomes

Was a primary endpoint defined or used in a power calculation? (Yes/No)

Type of primary endpoint eg. clinical, surrogate, other (categorical)

Type of other outcomes reported (categorical)

Was mortality a pre-defined endpoint? (Yes/No)

Was the primary endpoint found to be significant? (Yes/No)

If so, did it favour the investigational intervention group, if applicable? (Yes/No)

Was an intention-to-treat analysis performed? (Yes/No)

Was the trial stopped early? (Yes/No)

If so, reason stopped early (text)

Did the authors explicitly call for further research to answer *same* research question eg. larger trial? (Yes/No)

Trial publication reviewed by (initials of investigator)

Trial publication checked by (initials of investigator)

**References**

1 Lefebvre C, Manheimer E, Glanville J, The Cochrane Information Retrieval Methods Group. Searching for Studies. In: Higgins JPT, Green S eds. *Cochrane Handbook for Systematic Reviews of Interventions*. Wiley: Chichester, 2011. Available from [www.handbook.cochrane.org](http://www.handbook.cochrane.org) [accessed 15 February 2017]

2 Leclercq E, Leeflang MM, van Dalen EC, Kremer LC. Validation of search filters for identifying pediatric studies in PubMed. *J Pediatr* 2013; **162:** 629–34.

3 Manríquez JJ. A highly sensitive search strategy for clinical trials in Literatura Latino Americana e do Caribe em Ciências da Saúde (LILACS) was developed. *J Clin Epidemiol* 2008; **61:** 407–11.

4 Higgins JPT, Altman DG, Sterne JAC, The Cochrane Statistical Methods Group and the Cochrane Bias Methods Group. Assessing risk of bias in included studies. In: Higgins JPT, Green S, eds. *Cochrane Handbook for Systematic Reviews of Interventions*. Wiley: Chichester, 2011. Available from [www.handbook.cochrane.org](http://www.handbook.cochrane.org) [accessed 15 February 2017]
